# Children’s Gender Stereotypes in STEM Following a One-Shot Growth Mindset Intervention in a Science Museum

**DOI:** 10.3389/fpsyg.2021.641695

**Published:** 2021-05-10

**Authors:** Fidelia Law, Luke McGuire, Mark Winterbottom, Adam Rutland

**Affiliations:** ^1^Department of Psychology, University of Exeter, Exeter, United Kingdom; ^2^Faculty of Education, University of Cambridge, Cambridge, United Kingdom

**Keywords:** growth mindset intervention, implicit theories, gender stereotypes, STEM, informal science learning

## Abstract

Women are drastically underrepresented in science, technology, engineering, and mathematics (STEM) and this underrepresentation has been linked to gender stereotypes and ability related beliefs. One way to remedy this may be to challenge male bias gender stereotypes around STEM by cultivating equitable beliefs that both female and male can excel in STEM. The present study implemented a growth mindset intervention to promote children’s incremental ability beliefs and investigate the relation between the intervention and children’s gender stereotypes in an informal science learning site. Participants (*n* = 143, female *n* = 77, male *n* = 66, 5–12-years-old, *M*^age^ = 8.6, SD = 1.7) were visitors to a science museum who took part in an interactive space science show. Participants who were exposed to a growth mindset intervention, compared to the participants in the control condition, reported significantly less gender stereotyping around STEM by reporting equitably in the stereotype awareness measure. Relatedly, participants in the control condition reported male bias gender stereotype in the stereotype awareness measure. Further, children between 5 and 8-years-old reported greater male bias stereotypes awareness and stereotype flexibility in space science compared to children between 9 and 12-years-old. Lastly, children demonstrated in-group bias in STEM ability. Male participants reported gender bias favoring males’ ability in stereotype flexibility and awareness measures, while female participants reported bias toward females’ ability in stereotype flexibility and awareness measures. These findings document the importance of a growth mindset intervention in buffering against STEM gender stereotyping amongst children, as well as the significant role a growth mindset intervention can play within an informal science learning site.

## Introduction

Women are underrepresented in science, technology, engineering, and mathematics (STEM) careers (European Commission, 2019; WISE, 2019). In the United Kingdom, only 16% of computer science professionals and 10% of engineering professionals are women (WISE, 2019, 2020). Along with this gender disparity, the STEM workforce is facing a severe shortage in the number of skilled graduates required to meet the demand for STEM job vacancies (Bosworth et al., 2013; UKCES, 2014). One contributing factor that can help explain this gender disparity is gender stereotypes about STEM that associate “brilliance” in these fields with men more than women, which may undermine women’s willingness to pursue a career in STEM (Shapiro and Williams, 2012; Leslie et al., 2015; Meyer et al., 2015).

The present study examines the relation between a one-shot growth mindset intervention and children’s gender stereotypes about STEM ability in the context of space science – a highly male-dominated domain (Flaherty, 2018). Growth mindset taps into the beliefs that people’s intelligence and ability are malleable and are subject to change (Dweck, 2015), thus opposing gender stereotypical views that boys are innately smarter than girls and that girls might not have the same innate ability to do well in STEM domains.

Gender stereotypes about females’ ability in STEM can limit women’s future engagement in STEM careers. Specifically, studies show that women are underrepresented in areas where success is believed to require high intellectual abilities, as these abilities are associated with men more than women (Leslie et al., 2015; Meyer et al., 2015). These gender stereotypes contribute to the widening gender gap in STEM disciplines (National Science Foundation, 2013; Wang and Degol, 2017). From the age of six, children begin to show gender-stereotypical beliefs that boys are better than girls in some STEM subjects, such as programming (Master et al., 2017a). Within the same age group, children also believe that boys are in general smarter than girls (Bian et al., 2017). Considering the possibility that female disengagement in STEM may develop early, efforts to challenge gender stereotypical beliefs about STEM should focus on children from a young age. This is why the present study examines the effectiveness of a growth mindset intervention on gender stereotypes among children from the age of five to twelve in the context of an informal science learning site (ISLS; e.g., science centers, museums, zoos, and aquaria).

### Implicit Theories of Intelligence

The implicit theories of intelligence proposed by Dweck and Leggett (1988) posit that an individual’s beliefs or views about intelligence can influence how one approaches challenges, orientates goals, and responds to criticisms (Dweck, 2015). For people with a fixed view of intelligence (i.e., a fixed mindset), intelligence in a specific area is viewed as a fixed entity and innate ability with limited opportunity for growth. In contrast, people with a malleable view of intelligence (i.e., growth mindset) believe that one can get smarter through learning and work toward that as a goal for self-improvement (Dweck and Leggett, 1988). Relatedly, a person with a growth mindset focuses on the process of learning and developing their ability, while a person with a fixed mindset focuses on the end goal of validating personal ability (Dweck and Leggett, 1988; Gunderson et al., 2013). As such, individuals with growth mindsets react to difficulties with adaptive and helpful learning strategies such as persistently trying to answer difficult questions and set achievable goals, while people with fixed mindsets react with helplessness such as giving up on difficult learning tasks (Blackwell et al., 2007; Rattan et al., 2015).

A large body of literature shows that children’s implicit theories about intelligence can set them on very different trajectories of motivation and learning (Yeager and Dweck, 2012; Dweck, 2015; Rattan et al., 2015). Endorsing a growth mindset is positively related to academic performance in schools (Yeager et al., 2019), particularly for students facing learning difficulties (Blackwell et al., 2007; Claro et al., 2016). Further, in a longitudinal study that followed college students through a calculus course, researchers found that the more women perceived their college peers as having a malleable view on math ability, the more they felt a sense of belonging to math (Good et al., 2012). This sense of belonging, in turn, led to an increased desire to pursue math, even when the environments were perceived to be highly gender stereotypical. This study suggests that the perception of math ability as a malleable entity led to a heightened sense of belonging in a stereotypical male domain for women and buffered against the negative effects of gender stereotyping which include a decreased intention to pursue math. In this study, even in the face of negative stereotypes about females’ ability in math, female students maintained high intention to pursue math in the future, felt greater belonging and earned high grades in math when they endorse a growth mindset. However, the relationship between a growth mindset and gender stereotyping is unclear amongst children.

Arguably, beliefs that the brains of both boys and girls are capable of growth, and that intelligence can be developed by learning, should be related to more equitable beliefs about STEM gender abilities. One of the ways to test this prediction is to investigate the relation between a growth mindset intervention and children’s gender stereotypes in a specific STEM domain. The present study does this and aims to improve our understanding of how growth mindset is related to children’s STEM gender stereotypes.

### Gender Stereotypes

Developmental findings suggest that gender stereotyping in STEM emerges early. A recent study found children from 3 to 5 years of age endorsed strong gender stereotypes about STEM and found less support for counter-stereotypical STEM career options (Mulvey and Irvin, 2018). From the age of six, children believe that boys are better in robotics than girls (Master et al., 2017a). Within the same age group, children believe that boys are smarter than girls (Bian et al., 2017). It is important to note that gender differences cannot be seen in academic ability (Kovas et al., 2007), yet young children still exhibit gender stereotypical attitudes toward STEM career options and interests. This phenomenon is especially apparent in domains where gender inequality is seen in the working world. Data shows that men constitute more than 80% of the workforce in engineering and technology (WISE, 2019), which requires mathematics skills. Elementary school children reported boys liking mathematics more than girls (Cvencek et al., 2011), although this may not be reflected in grade attainment (Kovas et al., 2007). On that note, it is important to pay attention to children’s responses about whom they think *usually* does well in STEM. In other words, this is their awareness of gender stereotypes in STEM (Liben and Bigler, 2002), which is reflective of what children think the world is *currently* like. Further, it is also important to focus on children’s stereotype flexibility, as it reflects who they believe *can* do well and succeed in STEM (Liben and Bigler, 2002), which is reflective of what they believe the world *can* be like. In this study, we are interested to explore both stereotype flexibility and stereotype responses. Based on past studies, stereotype awareness and flexibility can be considered separately with children from age 4- to 11-year-old (Liben and Bigler, 2002; Trautner et al., 2005). In this study, we utilized stereotype awareness and flexibility measures by Liben and Bigler (2002) to investigate children’s knowledge of and beliefs about gender stereotypes. Furthermore, past studies demonstrate interesting gender differences in children’s gender stereotyping around STEM. In a recent study conducted at five ISLS, researchers found children from 5 to 8-years-old were more likely to report that members of their own gender group usually and can do well in STEM (McGuire et al., 2020). However, for older children (aged 8–11), boys were more likely to show in-group gender bias in gender stereotyping. Boys were significantly more likely to state that boys can do well when asked about STEM ability, whereas girls in this age range do not share the same in-group bias. Another study conducted in three countries with undergraduate students found a similar trend whereby men endorsed more male-favoring stereotypes than women, while women endorsed female-favoring stereotypes more than men (Moè et al., 2020). This effect was more pronounced in countries with a larger gender gap index in STEM. Put together, these findings demonstrate interesting gender differences observable across children throughout young adulthood, as well as pointing toward the critical window to intervene with promoting girls’ beliefs in their gender group’s ability to do well in STEM early in age.

Moreover, children in different developmental stages display varying levels of gender stereotypes around STEM. For example, research found younger children engage in more gender stereotyping than older ones (McGuire et al., 2020). Further, Moè (2018) found that children endorse gender stereotype beliefs from the age of eight, but these stereotypes did not relate to children’s performances in mathematics. Besides, children before 8 years of age show strong gender stereotypes around intellectual ability (Bian et al., 2017). This is because from 8 years of age, children transition from preoperational to concrete operational thought (Piaget, 1971). This means that at 8 years old or younger, children are less likely to perceive differences in ability between gender groups and pre-judge an individual based solely on their gender group membership and not any other characteristics they may display. However, research suggests that with age (from approximately 8 years), children show an age-related increase in the ability to process multiple classifications and, therefore, show more stereotype flexibility (Bigler and Liben, 2007; Martin and Ruble, 2010). The current research is interested therefore to explore the developmental differences between children before the age of eight and after. In theory, interventions targeting STEM ability may be more effective in challenging stereotypes beliefs among children below 8 years old.

Gender stereotypes are damaging to girls’ career aspiration and motivation (Reuben et al., 2014). Gender stereotypes also have the potential to impact other factors such as self-efficacy, identity, belonging, engagement, and persistence in STEM (Eddy and Brownell, 2016). Thus, to equalize the gender representation in STEM fields, research suggests that it might be necessary to go back to early school science education (Kerkhoven et al., 2016), as children’s gender stereotypes develop rapidly between the ages of 6 and 10 (McKown and Weinstein, 2003) and gender biases in STEM fields emerge early in age (Mulvey and Irvin, 2018; McGuire et al., 2020). However, so far, interventions have been limited to formal educational settings, such as schools. Young people also spend time engaging in informal learning outside of the formal education environment, such as science centers and museums.

In the United Kingdom, 5.3 million people visited five of the largest science museums in 2017 (Science Museum Group, 2018). International data from 181 museums and science centers worldwide documented that over 67 million people visited ISLS in 2016 (ATSC, 2016). Less work has been done, however, within these contexts to understand how, coupled with theory-based educational interventions, ISLS can be effective in challenging STEM gender stereotypes among young people. Research on growth mindset interventions have largely been conducted at formal educational settings (i.e., schools and universities); thus, the present study extends research on growth mindset interventions to ISLS.

### Growth Mindset Interventions

Interventions that communicate growth mindsets have effectively promoted students’ incremental beliefs about intelligence (DeBacker et al., 2018). Further, research on growth mindset interventions has also led to positive outcomes in students’ academic achievements and motivation in schools (Blackwell et al., 2007; Good et al., 2012; Paunesku et al., 2015; Yeager et al., 2019). Research shows that growth mindset interventions can be effectively executed through one-shot (single session) programs. These interventions can take place in schools or through online platforms (Blackwell et al., 2007; Yeager and Dweck, 2012; Rattan et al., 2015; Yeager et al., 2016; DeBacker et al., 2018; Burgasser, 2019).

Despite a mounting interest in growth mindset interventions, this approach has yet to be applied to informal learning contexts, as most research has been conducted at formal learning settings (Aronson et al., 2002; Blackwell et al., 2007; DeBacker et al., 2018). However, one line of research shows that growth mindset messages can be communicated through interactive educational video games (O’Rourke et al., 2016). In this study, a 3-min growth mindset message was related to higher persistence as children played more levels of the game after receiving growth mindset related feedback, as compared to children in the control condition. There is a need for research to examine the effectiveness of delivering a growth mindset intervention in ISLS.

### The Present Study

Therefore, in the current study, we uniquely partnered with ISLS practitioners in designing and examining the relation between a growth mindset intervention and children’s gender stereotypes around STEM. Here, a growth mindset intervention was delivered as part of an interactive space science show within a science museum. Growth mindset is a domain-specific construct (Dweck, 2015), so in this study, the focus is specifically on the domain of space science. Space science was selected as the STEM subject of study because women are drastically under-represented in this discipline and there is a higher dropout rate of women in space science as compared to men (Hill et al., 2010; Flaherty, 2018; Porter and Ivie, 2019).

This study focuses on children’s (age 5–12 years old) responses to who they think *can* do well (stereotype flexibility) and *usually* do well in space science (stereotype awareness). Children begin to categorize the world based on gender early in life (Quinn et al., 2002) and from 5-years of age they can segregate occupations by gender roles and place different values on traditionally masculine and feminine careers (Weisgram et al., 2010). The present study tested the relation between growth mindset intervention and gender stereotypical views around space science as well as to compare the developmental differences between children in middle childhood (age 5–8 years old) and children in late childhood (age 9–12 years old). We draw the same predictions for both stereotype awareness and stereotype flexibility.

### Hypotheses

H1: Children in middle childhood (8 years old or below) will report greater gender stereotyping by showing more male bias (i.e. favoring male ability over female ability) in space science ability compared to those in late childhood (9 years old or above).

H2: Children in the growth mindset condition will exhibit significantly less gender stereotyping in space science with more equitable responses, while children in the control condition will exhibit significant gender stereotyping by showing more male bias in space science.

H3: The growth mindset intervention will be particularly effective for children within middle childhood (8 years old or below). Specifically, children in middle childhood within the growth mindset condition will respond more equitably to gender stereotype measures compared to children in the control condition.

H4: Male participants will show in-group bias by reporting greater gender stereotyping in favor of male’s ability than reported by female participants.

## Materials and Methods

### Participants

One hundred and sixty-seven participants completed the study in a science center in the Midlands of the United Kingdom. Five participants who reported their gender as “other” were excluded from the analysis due to insufficient power to include the gender-other category in the analysis. In addition, two participants with no age information were excluded, and 17 participants older than 13-years were also excluded as the present study focused on middle and late childhood rather than adolescence. An *a priori* power analysis was conducted using G^∗^Power3 (Faul et al., 2007) to identify the total sample required to achieve a power of 0.80 using a two-tailed test with a medium effect size of 0.25. The results showed that a total sample of 128 participants was required. Altogether 143 participants were included in the analyses (female *n* = 77, male *n* = 66). Seventy-three participants (female *n* = 45, male *n* = 28) were in the growth mindset condition and 70 participants (female *n* = 32, male *n* = 38) were in the control condition. Participants were divided into two age groups: middle childhood (*n* = 75, *M*^age^ = 7.21, SD = 0.81, minimum = 5-years, maximum = 8-years) and late childhood (*n* = 68, *M*^*age*^ = 10.04, SD = 1.06, minimum = 9-years, maximum = 12-years). Overall, 77% of participants were White, 12% Asian, 3% mixed-race/dual heritage, and 8% chose not to disclose ethnicity. Parental consent and child assent were obtained for all participants.

### Procedure and Experimental Manipulation

All measures were approved by the Goldsmiths, University of London’s Ethics Committee as part of the project “Growth mindset intervention among children”. The protocol was completed in a science center following an hour-long interactive space science show. The space science show includes images of both male and female astronauts. When visitors were invited to take part in the show, both male and female visitors were invited at the same time. Participants in the experimental condition received a growth mindset intervention during the show. The intervention was adapted from “You Can Grow Your Intelligence” (designed by Mindsets Inc.; Blackwell et al., 2007) and tailored to fit the space science show at the science museum. The intervention conveyed a message about brain malleability and highlights how ability can be developed through persistent learning. It also frames setbacks and challenges as opportunities for learning and growth. The growth mindset message was delivered by either a female or a male voice accompanying a photograph of astronauts. Voice gender was varied in order to control for any influence of this factor on our dependent variables. Participants in the control condition experienced the same interactive space science show without hearing the growth mindset message below.

*“It was nearly an impossible task to send astronauts to the moon, but this year we are celebrating the 50th anniversary of this great achievement. This was all possible because all of us, including you, have an amazing brain that can develop and become smarter as you learn! You cannot see it, but each time you learn a new thing, the tiny connections in your brain multiply and get stronger. The more you challenge yourself to learn, the more your brain will develop and grow. Just like children, who first don’t know how to read, but after learning and practising and making many mistakes (just like the video of the astronauts we watched, learning how to walk on the moon and keep falling) they can eventually learn how to read! The baby’s brain has now changed, it has gotten smarter. Like a muscle that grows when we exercise, our brain grows smarter when we keep learning and trying! Especially when you are learning a difficult subject or trying a challenging task, these are the perfect opportunity for your brain to grow and be stronger! Would you like to strengthen your brain and be smarter? You can! At whatever age you are, your brain develops and become stronger when you learn new things.”*

The intervention as a whole consisted of three elements: a growth mindset message, a writing task, and a manipulation check at the end of the survey. The writing task asked participants to write a short message of encouragement to a friend who is struggling to learn about space science (adapted from DeBacker et al., 2018). This writing task is rooted in getting participants to advocate for a particular position (here, growth mindset beliefs in learning space science), a phenomenon called the “saying-is-believing effect” (Higgins and Rholes, 1978). At the end of the survey, participants answered two manipulation check items (e.g., “According to the show, what happens to our brain when we learn new things?”). All participants answered at least one manipulation check question correctly and were therefore included in the analyses presented below. Children who participated in this study received a gift bag worth £5 in exchange for completing the survey. All participants were part of family groups visiting the science center, consisting of at least one adult and one child.

### Gender Stereotypes Measures

The gender stereotype measure was adapted from Liben and Bigler (2002) to assess children’s stereotype awareness and stereotype flexibility. Participants read a series of sentences and marked on a line to indicate their agreement with the sentence from 0 = not true at all to 100 = very much true with a slider marked in increments of 10. For *Stereotype Awareness* the items are “I think that girls usually do well in space science” and “I think that boys usually do well in space science”, whereas, for *Stereotype Flexibility*, the items are “I think that girls can do well in space science” and “I think that boys can do well in space science”. Using these measures, male bias score for stereotype awareness and stereotype flexibility were created by subtracting the response to the question about girls from the response to the question about boys. The male bias score scaled from −100 (maximum female bias = participant responded 100 to girls’ question and 0 to boys’ question) to 100 (maximum male bias = participant responded 100 to boys’ question and 0 to girls’ question) and as the mid-point of the scale, zero score indicates an equitable gender stereotype response.

### Data Analytical Strategy

To observe the differences in children’s gender stereotyping based on their age, gender, and experimental conditions, we conducted a 2 (Age; Middle Childhood, Late Childhood) × 2 (Gender; Female, Male) × 2 (Mindset condition; Growth mindset, Control) independent ANOVA with male bias stereotype awareness and male bias stereotype flexibility as the dependent variable respectively using SPSS Version 25 (IBM Corp, 2018). Where appropriate, simple main effects comparisons were conducted using Bonferroni corrections for multiple comparisons. To test the direction of gender bias in a given condition, we carried out one-sample *t-*tests to determine whether the mean of a given group (i.e. middle childhood) differed significantly from the criterion value of zero (i.e. no bias toward male or female ability).

## Results

### Stereotype Awareness

Consistent with H1, a significant main effect of age was observed, *F*(1,133) = 8.03, *p* = 0.005, $\eta_{\text{p}}^{2}=0.06$ Participants in middle childhood reported significantly greater male bias (*M* = 11.12, SD = 28.74) compared to participants in late childhood (*M* = −2.69, SD = 29.07). Responses in middle childhood [*t*(72) = 2.39, *p* < 0.05, *d* = 0.28] differed significantly from the mid-point of the scale, in the direction of male bias. In contrast, responses in late childhood did not differ from the mid-point of the scale [*t*(67) = 1.09, *p* > 0.05, *d* = 0.13].

**FIGURE 1:**
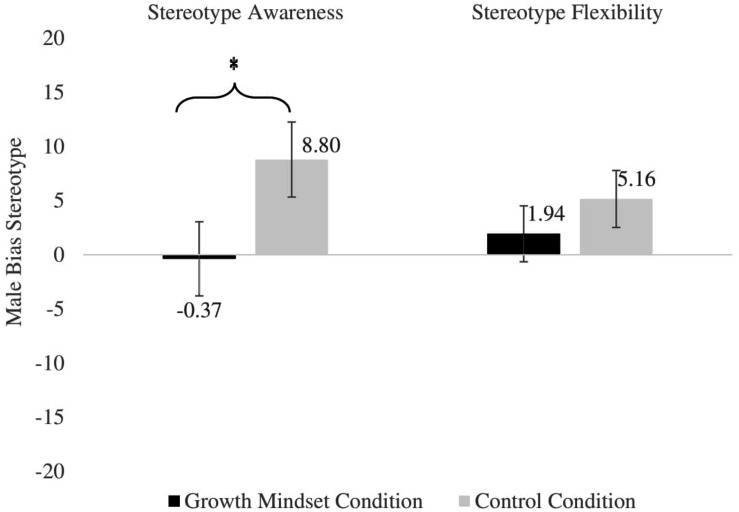
Male bias stereotype as a function of stereotype measure and experimental condition (scores above zero indicate response in favor of male’s ability, zero scores indicate equitable response and below zero indicate in favor of female’s ability; error bars represent standard error of the mean). Stereotype awareness measures who children believe *usually* do well in space science while stereotype flexibility measures who children believe *can* do well in space science. ^∗^Denotes *p* = 0.06.

Further, in line with the second hypothesis, the analysis yielded a marginally significant effect of growth mindset, *F*(1,133) = 3.54, *p* = 0.062, $\eta_{\text{p}}^{2}=0.03$ (Figure 1). In partial support of H2, participants in the control condition reported greater male bias (*M* = 8.80, SD = 29.25) than participants in the growth mindset condition (*M* = −0.37, SD = 28.59). Further analysis revealed that responses in the control condition differed significantly from the midpoint of the scale in favor of male bias [*t*(67) = 2.57, *p* < 0.05, *d* = 0.31]. In contrast, participants in the growth mindset condition did not differ from the mid-point of the scale [*t*(72) = −0.64, *p* > 0.05, *d* = 0.07]. Hypothesis 3 was not supported as no significant interaction effects were observed between mindset condition and age, *F*(1,136) = 0.002, *p* = 0.97, $\eta_{\text{p}}^{2}=0.001$, indicating that the effect of the mindset intervention was the same for both age groups. Results did not support the prediction that the intervention would be particularly effective for children within middle childhood (8 years old or below).

In support of H4, a main effect of gender was observed, *F*(1,133) = 17.89, *p* = 0.001, $\eta_{\text{p}}^{2}=0.12$. Male participants reported male in-group gender bias (*M* = 14.52, SD = 28.69) while female participants reported female in-group bias (*M* = −6.09, SD = 31.47). Male participants’ responses differed significantly from the midpoint of the scale in favor of male ability [*t*(63) = 3.80, *p* = 0.001, *d* = 0.47] and female participants’ responses differed significantly from the midpoint of the scale in favor of female ability [*t*(76) = −2.17, *p* = 0.03, *d* = 0.47). There were no significant interactions between participants’ gender and the mindset conditions, *F*(1,137) = 0.02, *p* = 0.97, $\eta_{\text{p}}^{2}=0.01$. The means and standard deviations for stereotype awareness response by mindset condition age, and gender are included in Table 1.

**TABLE 1:** Stereotype awareness response difference score by mindset condition, age, and gender.

		**Middle childhood**		**Late childhood**		**Total**
		**Male**	**Female**	**Male**	**Female**	
Growth mindset condition	*M*	18.67	−4.70	0.31	−15.78	−2.34
	SD	37.94	23.89	13.18	35.19	31.54
Control condition	*M*	30.82	−0.33	8.26	−3.57	9.19
	SD	38.94	24.12	22.28	16.10	29.51
Total	*M*	9.45		−3.47		
	SD	33.80		26.35		

### Stereotype Flexibility

Consistent with H1, a significant main effect of age group was observed, *F*(1,132) = 8.57, *p* = 0.004, $\eta_{\text{p}}^{2}=0.06$. Participants in middle childhood reported greater male bias (*M* = 8.93, SD = 21.82) than participants in late childhood (*M* = −1.84, SD = 21.91). Responses in middle childhood differed significantly from the mid-point of the scale, in favor of male ability [*t*(71) = 2.3, *p* < 0.05, *d* = 0.27], while responses in late childhood did not differ from the mid-point of the scale [*t*(67) = −0.81, *p* > 0.05, *d* = 0.10].

We did not observe a significant main effect of mindset condition on stereotype flexibility, *F*(1,132) = 0.56, *p* = 0.38, $\eta_{\text{p}}^{2}=0.006$. No significant difference was observed between participants in the control condition (*M* = 1.94, SD = 22.05) and participants in the growth mindset condition (*M* = 5.16, SD = 21.54). Participants’ responses in the control condition [*t*(66) = 1.74, *p* = 0.09, *d* = 0.21] and participants’ responses in the growth mindset condition [*t*(72) = 0.167, *p* = 0.87, *d* = 0.02] both did not differ from the mid-point of the scale. Thus, H2 was not supported by the analyses on stereotype flexibility. Further, H3 was not supported as there were no interaction effects between mindset condition and age group, *F*(1,132) = 2.52, *p* = 0.12, $\eta_{\text{p}}^{2}=0.019$. Particularly, results did not show, as had been predicted, that the intervention will be particularly effective for children within middle childhood (8 years old or below).

Lastly, a main effect of gender was observed as predicted in H4, *F*(1,132) = 22.40, *p* = 0.001, $\eta_{\text{p}}^{2}=0.15$. Male participants reported greater male in-group gender bias (*M* = 12.27, SD = 21.62) while female participants reported greater female in-group bias (*M* = −5.18, SD = 22.0). Male participants’ responses differed significantly from the midpoint of the scale in favor of male ability [*t*(63) = 4.05, *p* = 0.001, *d* = 0.51] and female participants’ responses differed significantly from the midpoint of the scale in favor of female ability [*t*(75) = −2.23, *p* = 0.03, *d* = 0.26]. There were no significant interactions between participants’ gender and mindset conditions, *F*(1,136) = 2.97, *p* = 0.60, $\eta_{\text{p}}^{2}=0.01$. The means and standard deviations for stereotype flexibility response by mindset condition age, and gender are included in Table 2.

**TABLE 2:** Stereotype flexibility response difference score by mindset condition, age, and gender.

		**Middle childhood**		**Late childhood**		**Total**
		**Male**	**Female**	**Male**	**Female**	
Growth mindset condition	*M*	14.53	−5.47	4.0	−5.05	0.37
	SD	27.24	15.55	8.72	14.71	18.95
Control condition	*M*	25.64	1.29	4.89	−11.21	5.88
	SD	34.48	24.14	12.91	24.77	27.64
Total	*M*	7.56		−1.81		
	SD	27.85		16.93		

## Discussion

The present study found, as predicted, that participants who were exposed to a growth mindset intervention compared to the participants in the control condition reported significantly less gender stereotyping around STEM, by demonstrating less male bias in the stereotype awareness measure. However, no difference was observed between participants who experienced a growth mindset intervention and participants in the control condition for stereotype flexibility measure. Participants in both conditions responded equitably. The findings also showed how those children between 5 and 8-years-old reported greater male bias stereotypes awareness and stereotype flexibility in space science compared to children between 9 and 12-years-old. Further, children demonstrated in-group bias for their own gender group. Male participants reported greater bias favoring males in stereotype flexibility and awareness measures, while female participants reported greater bias toward females in stereotype flexibility and awareness measures.

The present research makes two novel contributions to the literature. First, the findings of the study demonstrate a relation between a one-off growth mindset intervention and children’s gender stereotypes awareness in the domain of space science. Secondly, the present study extends previous growth mindset interventions research by demonstrating how a growth mindset intervention can be executed in an interactive science show at an informal learning setting, such as a science museum.

Examining gender stereotypes about space science across children in middle childhood and late childhood, we observed that younger children reported greater male bias in stereotype awareness and stereotype flexibility compared to older children. The lack of male bias in gender stereotype in STEM ability is consistent with prior studies investigating stereotypes around math and science ability among children in late childhood (Muzzatti and Agnoli, 2007; Kurtz-Costes et al., 2014). The present finding is also consistent with recent research conducted at ISLS in the United Kingdom and the United States, which documented greater male bias stereotypes around STEM with younger children compared to their older counterparts (McGuire et al., 2020). Efforts to challenge these stereotypes should begin early with children in middle childhood as evidenced in the present study.

An important contribution of the current study was that we investigated how growth mindset intervention in an ISLS relates to children’s male bias stereotypes in the male dominated domain of space science. Specifically, the present study found mindset intervention a buffer against STEM gender stereotyping in some ways. In this study, we explicitly measured children’s stereotype awareness to elicit *knowledge* of gender stereotypes, and children’s stereotype flexibility to elicit *attitudes* toward stereotypes (Signorella et al., 1993; Liben and Bigler, 2002). Notably, we found that in the growth mindset intervention condition, children reported equitable responses to the stereotype awareness measure, as compared to children in the control condition who responded with greater male bias. Although the effect size was small, these findings indicate that the understanding of brain malleability is associated with more equitable responses for both boys’ and girls’ understanding of who usually does well. This is an interesting finding as national statistics show that space science-related careers including astronomy are highly male-dominated, suggesting perceived male superiority in these careers (Cesarsky and Walker, 2010; Porter and Ivie, 2019), yet we observed that a growth mindset message is associated with more equitable stereotype awareness responses. This finding has promising implications because when children believe that both boys and girls *usually* do well in space science, both gender groups should be likely to engage in space science-related studies or activities in the future. This is especially important for girls as they tend not to engage in STEM activities that they view as not suitable for them or that they cannot do well in (Bian et al., 2017; Master et al., 2017a).

We did not find the same relation between mindset intervention and stereotype flexibility. Notably, children in both conditions responded equitably on this measure, indicating that they believe both boys and girls *can* do equally well in space science. Since this measure elicits children’s attitudes toward stereotypes (Signorella et al., 1993), this finding suggests that children were less willing to show gender biased attitudes toward space science ability explicitly. One possible explanation is that this research was conducted in an interactive space science show that was facilitated by both male and female ISLS educators and throughout the show, both boys and girls had equal chances to take part in space science activities during the show. ISLS often encourage boys’ and girls’ involvement in STEM to promote interest and engagement of all (National Research Council, 2010) which may in turn be related to more equitable beliefs about who succeed in these areas.

Another possible explanation for the equitable responses in both control and growth mindset condition could be that children are less inclined to explicitly report stereotypical *attitudes* toward boys’ and girls’ ability. In contrast, when the measure was less directive as it taps on their *knowledge* of the gender representation in STEM (i.e., who *usually* do well) children are more likely to demonstrate gender biases. This could be due to social desirability artifacts and that children may respond in ways which they believe are more socially desirable or acceptable, especially when they are answering the questions in the presence of their family members at the space science show. On that note, it would be interesting to look at how children respond to gender stereotype endorsement using implicit stereotype measures at ISLS. With regards to a socially sensitive domain such as gender stereotypes, utilizing implicit stereotype measures might be recommended (Hofmann et al., 2005; Greenwald et al., 2009; Cvencek et al., 2011).

Furthermore, the present findings show participants’ gender played a role in their gender stereotype responses independent of the experimental condition and age. Both male and female participants reported significant in-group bias in awareness and flexibility measures. This is consistent with recent research (McGuire et al., 2020) and the developmental literature pertaining to children’s strong support for their ingroup which may sometimes result in manifestations of biases against other groups (Bigler and Liben, 2007). These findings present important implications for practice. Practitioners could consider ways to leverage on female in-group biases to foster a strong interest in STEM and cultivate a sense that females, along with males, can all do well in STEM activities.

One way to enhance these beliefs is by organizing group activities for female visitors. Past studies demonstrate that a sense of social group membership (boys and girls mixed group) can enhance children’s persistence on a STEM task, and increase interest and perceived self-efficacy in the STEM task (Master et al., 2017b). Encouraging girls to participate in a STEM activity together, for instance, having a female science activity group to take part in a stereotypically male activity (e.g. build a car engine), may foster female’s interest in these activities. Besides that, more work should consider the impact of male in-group bias in STEM and how this can cultivate boys’ interest without hindering girls’ engagement. More research in these areas is important because children’s perception of who is able to do well in STEM has a significant impact that lasts for a lifetime as it directly influences their educational and career engagement in the future (Francis, 2000; Davies et al., 2005; Cheryan et al., 2011).

The present research breaks new ground by demonstrating how growth mindset intervention can be effectively executed at ISLS. The findings of the present study show that mindset interventions can be successfully carried out in a science museum with the collaboration between researchers and practitioners. This research-practice partnership offers many opportunities to explore research questions, test novel educational interventions, and design and implement impactful theory-based and outcome-focused practice (Anderson and Shattuck, 2012; Mulvey et al., 2020).

### Limitations and Future Directions

Future research should aim to examine how growth mindset messages relate to adolescents’ gender stereotypes in STEM ability. Adolescence is a crucial stage where there is a developmental decline in engagement and attitudes toward science (Osborne et al., 2003), especially among female teenagers in male-dominated areas such as engineering (Sadler et al., 2012). These developmental ages are pivotal moments to challenge stereotypes, and promote STEM interest and engagement. Given the relation between the growth mindset intervention and stereotypes in the present study, future work could explore how mindset interventions in informal learning sites may buffer against the negative effects of stereotype threats when gender identity is made salient among adolescents. Past studies show that in formal educational settings such as universities and schools, growth mindset messages are associated with less stereotype threat for minority status groups in academic attainment (Aronson et al., 2002). Yet, less is known about the relation between growth mindset and stereotype threat in STEM among children and adolescents in the context of informal learning.

Further, it is not clear whether the gender of the voice delivering the intervention may have been related to different effects in children’s stereotypical beliefs about STEM ability. In the present study, the voice of the growth mindset was treated as a control, whereby half of the participants heard the intervention delivered by a female voice and half by a male voice. Future research can build on this to investigate the effect of gendered growth mindset messages on children’s gender stereotype beliefs. Moreover, less is known about how the growth mindset message and gender stereotypes in STEM ability may reciprocally influence each other over time. A longitudinal research design would shed light onto how experiencing growth mindset intervention at ISLS relate to children’s gender stereotypes, attitudes and engagement around STEM. Prior growth mindset intervention research has shown promising long-term impact of mindset intervention with young people’s academic attainment and motivation (Yeager et al., 2019), but the relationship between mindsets and gender stereotypes in the long run is unclear. We found small effect sizes for the effectiveness of the one-shot mindset intervention. Future research can explore the possible effects of more than one shot intervention with a follow up intervention design over time.

Finally, the growth mindset intervention was conducted in a space science show at a science museum. It will be important for future research to examine the pattern of transfer of this mindset intervention into other areas such as children’s performance in school. Moreover, the current research explored the domain of space science specifically, while other interventions have investigated growth mindset messages in other STEM domains such as chemistry (Fink et al., 2018) and mathematics (Boaler et al., 2018). Less is known about the whether the effectiveness of growth mindset interventions transfers from one STEM domain to another. An important direction for future research is to investigate the transfer of the effects of growth mindset intervention from one STEM domain to another.

## Conclusion

For the first time, the current study examined the relation between delivering a one-off growth mindset intervention in an interactive space science show and children’s gender stereotypes. The findings demonstrate that knowledge about the malleability of ability is associated with more equitable gender stereotype awareness around STEM. The application of implicit theories in ISLS can play a role in children’s gender stereotype beliefs about STEM ability, which are known to be instrumental in the rising gender disparity between men and women in STEM (Sadler et al., 2012; Legewie and DiPrete, 2014). In our mission toward a more equitable STEM future, more research needs to be done to understand how to challenge children’s gender stereotyped beliefs about STEM ability from a young age. ISLS offer vibrant and dynamic activities aimed to increase engagement, interest, and motivation in STEM (National Research Council, 2010); thus, providing valuable opportunity to advance developmental science research around STEM and exciting platforms to develop research-based interventions for the public.

## Data Availability Statement

The raw data supporting the conclusions of this article will be made available by the authors, without undue reservation.

## Ethics Statement

The studies involving human participants were reviewed and approved by the Goldsmiths, University of London. Written informed consent to participate in this study was provided by the participants’ legal guardian/next of kin.

## Author Contributions

FL designed the study, developed the hypotheses, performed the data collection, analyzed the data, and drafted the manuscript. LM participated in the study design and performed data collection, and helped to draft the manuscript. MW participated in the study design and helped to draft the manuscript. AR supervised the study design, oversaw the development of the hypotheses and statistical analyses, and helped to draft the manuscript. All authors read and approved the final manuscript.

## Conflict of Interest

The authors declare that the research was conducted in the absence of any commercial or financial relationships that could be construed as a potential conflict of interest.

## Abbreviations

ISLS: informal science learning sites
STEM: science technology, engineering and mathematics
